# PreS1 Containing HBc VLPs for the Development of a Combined Therapeutic/Prophylactic Hepatitis B Vaccine

**DOI:** 10.3390/microorganisms11040972

**Published:** 2023-04-08

**Authors:** Andris Dishlers, Ivars Petrovskis, Dace Skrastina, Ieva Zarina, Ilva Lieknina, Juris Jansons, Inara Akopjana, Jelena Zakova, Velta Ose, Irina Sominskaya

**Affiliations:** Latvian Biomedical Research and Study Centre, Ratsupites Str. 1, 1067 Riga, Latvia

**Keywords:** HBV, HBc, virus like particles, immunogenicity, packaging

## Abstract

The available HBV vaccines based on the HBV surface protein are manufactured in yeasts and demonstrate excellent prophylactic but no therapeutic activity and are thus ineffective against chronic HBV infection. Five different HBV core proteins (HBc)—full length and C-terminally truncated—were used for the insertion of the short, preS1,aa 20–47 and long, preS1phil, aa 12–60 + 89–119 fragments. Modified virus-like particles (VLPs) were compared for their biotechnological and immunological properties. The expression level of HBc-preS1 proteins was high for all investigated proteins, allowing us to obtain 10–20 mg of purified VLPs from a gram of biomass with the combination of gel filtration and ion-exchange chromatography to reach approximately 90% purity of target proteins. The immunogenicity of chimeric VLPs was tested in BALB/c mice, showing a high anti-preS1 response and substantial T-cell proliferation after stimulation with HBc protein. Targeted incorporation of oligonucleotide ODN 1668 in modified HBc-preS1 VLPs was demonstrated.

## 1. Introduction

Despite the effective immunization of newborns with the marketed HBV surface protein (HBs)-based vaccines, some completely vaccinated adults do not reach dependable protection and 5 to 10% of infants of highly viremic pregnant women become chronically infected despite immediate after-birth immunization [1]. The rate of HB non-responders to existing vaccines remains high in groups of aged people, overweight people, medical workers, patients with renal insufficiency and on dialysis, patients after transplantation, patients with HIV as well as travelers to HBV-endemic regions [2]. Interferon therapy or treatment with nucleos(t)ide analogs suppresses HBV replication and decreases the development of cirrhosis, liver failure, hepatocellular carcinoma (HCC), and death, but is not able to eliminate the virus [3]. Thus, the remaining problems with hepatitis B include the inability to achieve a complete cure for chronic HBV infections, with the potential reactivation of HBV, as well as limited protection against HBV escape mutants.

Impressive efforts have been directed to improve existing HBV vaccines (1) by including preS1 and/or preS2 epitopes in the HB-based vaccine to enhance its prophylactic effect, and (2) through the combination of both structural proteins of HBV—the HBs and the HBc—in one formulation to aid the therapeutic effect of the vaccine. The combined protein–DNA vaccines have been developed in parallel as an alternative immunization strategy to achieve the goal of a universal HBV vaccine [4,5].

With epitope mapping of the HBV preS1 region, virus-neutralizing epitopes within preS1 sequences 19–26 and 37–45 were identified [6]. Within the preS1 region of HBV, aa - 13-59 can induce virus-neutralizing antibodies in mice [7]. The pre-S1 monoclonal antibody (mAb) used in this study (MA18/7) recognizes preS1 epitope DPAFR (aa 31–35) [8,9,10], and can inhibit the infection of primary Tupaia hepatocytes with HBV [11].

Available in several countries, Sci-B-Vac™ vaccine (SciVac Israel, Rehovot, Israel) containing the preS1 and preS2 fragments in addition to the HBs protein and produced in the mammalian cell line enabled a robust immune response against HBV infection during the phase III trial [12,13] and remarkably high anti-HB response (>100 mIU/mL) in 20 of 21 non- or low responders [14].

Due to its unique T- and B-cell immunogenicity, recombinant HBc was investigated as a promising therapeutic antigen against HBV—the first real trials to protect chimpanzees against HBV with the HBc VLPs were performed more than 30 years ago [15,16,17]. In a model with woodchuck hepatitis virus (WHV), it was shown that core protein (WHc) is a necessary and sufficient agent to protect woodchucks against WHV infection [18,19,20]; remarkably, the woodchucks were protected not only with the WHc, but also with the HBc vaccination [19]. Moreover, immunization of woodchucks with plasmids expressing both WHc and WHs efficiently suppressed WHV infection [21,22]; and later, the differences featured by the immunization of mice with the DNA or protein prototypes of the WHc vaccine were evaluated [23]. Furthermore, vaccination with whole cells expressing duck hepatitis B virus (DHBV) core (DHBc) protein was able to resolve chronic DHBV infection [24]. Recently, Boudewijns et al. proposed a novel therapeutic HBc vaccine that induced a strong polyfunctional cytotoxic T-cell response in mice using yellow fever vaccine as a live-attenuated vector for the expression of the HBc gene [25]. The plant-produced HBc VLPs were also presented as real prototypes of therapeutic vaccines [26,27]. An exhaustive study in a Tupaia model dealt with possible formulations of the intranasal HBc/HBs-based therapeutic vaccine [28].

Ulrike Protzer’s team, together with Rhein Biotech (Düsseldorf, Germany), contributed to the use of HBc as a therapeutic component in the HBV vaccine [4,29,30]. These activities resulted in the therapeutic vaccines TherVacB [4] and DV-601 (Dynavax Technologies, Emeryville, CA, USA) which combined HBs and HBc [29]. In parallel, a combination of the prospective combined HBc/HBs vaccine with an immune-stimulating CpG adjuvant was presented [30]. NASVAC, the therapeutic nasal HBc/HBs vaccine, was designed in Cuban laboratories [31,32,33,34] and underwent a phase III clinical trial for chronically infected hepatitis B patients [34].

**Figure 1:**
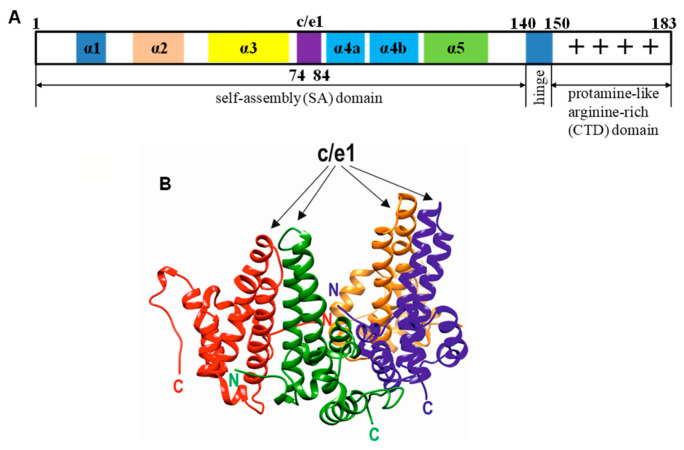
The structure of the HBc protein. (**A**) HBV HBc monomer represented by 183 aa-long HBc, genotype D1, subtype ayw2 (GenBank accession number XO2496) [47]. (**B**) The structure of two HBc dimers. Monomer chains A, B, C, and D are marked in red, green, blue, and orange, respectively. Surface-exposed loops with c and e1 epitopes are shown with arrows, N- and C- termini are labeled with N and C letters, respectively. Data were downloaded from the VIPERdb v3.0 (http://viperdb.scripps.edu, accessed on 1 May 2022) and visualized using UCSF Chimera Version 1.16 software [48].

HBc consists of 183 or 185 amino acid residues (aa), depending on the genotype [35]. The primary structure of the core protein can be divided into two domains, namely, the N-terminal self-assembly (SA) domain (aa 1–140) and the C-terminal RNA-binding protamine-like arginine-rich domain (CTD) (aa 150–183) [36] (Figure 1A). These domains are separated by the hinge peptide 141-STLPETTVV-149, which performs morphogenic functions [37]. The SA domain possesses a set of variable and conserved stretches that correspond to B-cell epitopes and structural elements, respectively, whereas the CTD and hinge peptide are the most conserved HBc regions without immunological importance (for reviews, see [38,39]). Four arginine blocks function as nucleic acid binding sites within the CTD [40]. HBc capsids that have been self-assembled from the truncated HBc proteins lacking the CTD, so-called HBcΔ variants, are not able to incorporate nucleic acids [41].

The HBc monomer is formed by five alpha helices connected by loops, where the hairpin of helices 3 and 4 dimerizes with the next monomer to form the central helical bundle. The major immunodominant region of the HBc antigen (MIR) is located within the SA domain (aa 78–82) on the tips of the HBc spikes [42] within B-cell epitopes *c* (HBc epitope) and *e1* (HBe epitope 1) [43,44] (Figure 1B). The MIR has been extensively used for the exposure of foreign immunogenic sequences (epitopes) on the HBc VLP surface and therefore provides the most efficient immunogenic activity (for reviews, see [45,46]).

Many laboratories around the world have successfully produced HBc VLPs in plants, insect, and mammal cells (for a review, see [38,39,49,50]). HBc variants from different genotypes have been produced and their corresponding VLPs purified by our group using *E. coli* [51,52,53,54] and yeast *P. pastoris* as expression systems [55].

It was found that the presence of nucleic acids is essential for the enhanced immunogenicity of the VLPs. The adjuvant effect of the bacterial RNA bound to the arginine-rich C-terminal domain of HBc was shown first by the immunization of mice with the HBc VLPs together with other proteins including HBs by the Reinhold Schirmbeck’s team [56,57]. Later, this group clearly demonstrated that particle-bound mammalian RNA functioned as TLR7 ligand and induced a Th1-biased humoral immunity in B6 but not in TLR7−/− mice [58,59]. Therefore, both endogenous bacterial and mammalian RNAs functioned as a natural adjuvant, facilitating the priming of Th1-biased immune responses. Later, the use of VLP as nanocontainers for genes and/or immunostimulatory oligonucleotide sequences was developed in detail by Strods et al. [53].

In the current study, we modified the surface of HBc VLPs by exposure of selected HBV preS1 epitopes to combine in one platform efficient anti-preS1 immunogenicity and T-cell immunogenicity provided by the T-cell epitope-rich HBc antigen. In parallel, the impact of the length of the CTD domain of HBc on recombinant HBc-preS1 VLP formation, immunogenicity, and their biotechnological properties was investigated. This approach was realized by our group before, first, by insertion of the hydrophilic stretch of the preS1 sequence into the MIR of unmodified HBc [60,61] or into the C-terminus of modified HBc HBc [62]; in both cases, the surface exposure of the inserts was achieved. It is worth mentioning that both of these modified VLPs provided highly efficient induction of adequate anti-HBV B- and T-cell responses.

## 2. Materials and Methods

### 2.1. Bacterial E. coli Strains

*Escherichia coli* (*E. coli*) strain RR1 [F^−^· r_B_^−^ m_B_^−^ leuB6 proA2 thi-1 araC14 lacY1 galK2 xyl-5 mtl-1 rpsL20 (Str^r^) glnV44 Δ (mcrC-mrr)] was used for the cloning and selection of recombinant plasmids. For the expression of HBc-preS1 genes, *E. coli* strains K802 (F^−^ r_K_^−^ m_K_^+^ e14 McrA metB1 lacY1 [or lacI-Y6] galK2 galT22 glnV44 mcrB) and BL21 [F^−^ ompT hsdS _B_ (r_B_^−^ m_B_^−^) gal dcm lon] were used.

### 2.2. Design of HBc-preS1 Proteins and Expression Plasmids

**Figure 2:**
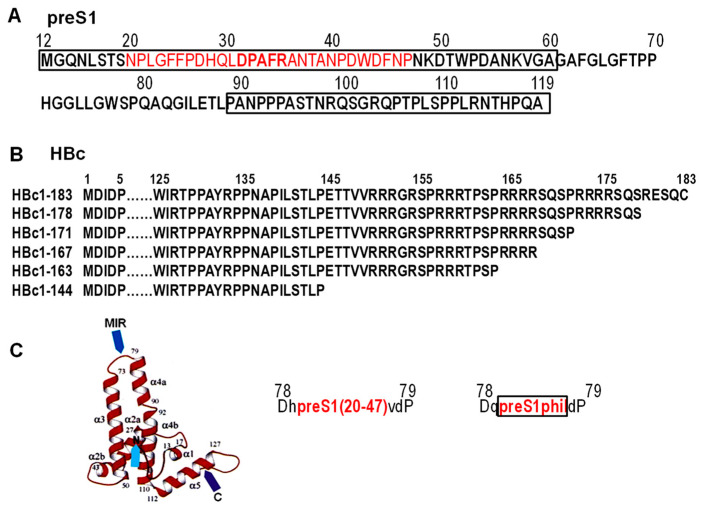
Structural elements used to design HBc-preS1 VLPs. (**A**) Structure of the preS1 region of HBV genotype D, and numeration of preS1 aa is according to the preS1 of HBV genotype A (GenBank accession number X70185); note: preS1 of genotype D is shorter by 11 aa than pres1 of genotype A. Short insertion fragment 20–47 is shown in red, and hydrophilic parts of the preS1 sequence spliced to create the long preS1phil fragment are shown in two boxes. Epitope DPAFR recognized by mAb MA18/7 is highlighted in bold red. (**B**) Full-length and truncated HBc vector variants were used for the insertion of preS1 fragments. Not shown aa of HBc are dotted. (**C**) Localization of surface-exposed MIR of HBc and aa surrounding the preS1 insertions: capital letters show aa of the HBc vector and small letters—linker aa added at the construction of expression plasmids. The numeration of aa in (**B**,**C**) is according to HBV genotype D (GenBank accession number XO2496).

Plasmids for the expression of chimeric HBcΔ-preS1(20–47) and HBcΔ-preS1phil proteins were described earlier [61,62], and these plasmids served here as a source for the amplification of corresponding preS1 containing sequences. HBcΔ refers here to C-terminally truncated, 144 aa-long HBc as a carrier protein, preS1(20–47) for the 28 aa long, and preS1phil for the 79 aa-long fragment linking together two hydrophilic fragments—aa 12–60 and aa 89–119—of the preS1 region of the LHBs protein of HBV genotype D [47]; aa numeration is according to HBV genotype A (Figure 2A). The source of HBV genes was the plasmid pHB320 with the full HBV genome (genotype D1, subtype ayw2) cloned in our lab (GenBank accession number X70185) [47]. In all our constructs the MIR of HBc was a target site for preS1 insertions (between aa D78 and P79). Corresponding PCR fragments were obtained using upstream primer 5′-GCCTCTAGATAACGCCTCAGCTCTGTATCG-3′ and downstream primer 5′-ACGAACAACAGTAGTCTCCGGAAGTGTTGATAAGATAG-3′. Primers were designed for the amplification of the HBc part (30–150 aa) that contained preS1 insertions in MIR and restriction sites *XbaI* and *Kpn2I* for further cloning. A gel extraction kit (Fermentas UAB, Vilnius, Lithuania) was used for the isolation of PCR fragments from agarose followed by incubation with *Xba*I and *Kpn*21 endonucleases at 37 °C for 1 h. For cloning of preS1 fragments, *Xba*I/*Kpn*21-treated appropriate HBc expression vectors [52] based on the pBR327 plasmid [63] were used. The expression of HBc-preS1 proteins in our constructs is under the control of the *E. coli* pTrp promoter [64]. Several HBc variants—full-length HBc (aa 1–183) and four truncated HBc proteins ending at aa positions 163, 167, 171, 178—were used as VLP carriers for the described preS sequences. Altogether, ten HBc-preS1 fusion proteins were designed (Figure 2B). In the designation of fusion proteins, the HBc vector is shown first, followed by the appropriate preS1 fragment—for example, 183preS1phil refers to the full-length HBc carrying the long preS1phil fragment. After *E. coli* transformation, three clones for each construct were analyzed for the presence of designed recombinant plasmid and plasmid structures were verified by sequencing (Figure 2).

### 2.3. Monoclonal Antibodies

Mouse anti-HBc mAb 13C9, recognizing epitope 134-PPNAPIL-140 [65] of HBc protein, and mouse anti-preS1 mAb MA18/7, recognizing the DPAFR subtype-independent linear 31-DPAFR-35 epitope [8,9,10], were used in this study.

### 2.4. Cultivation of Recombinant E. coli Cells and Purification of HBc-preS1 Proteins

Cultivation of *E. coli* cells for the expression of HBc-preS1 proteins and purification of target VLPs was performed essentially as described before [54] using Trp-rich phosphate-buffered 2xTY (2TYP), or Trp-deficient standard minimal M9 medium supplemented with casamino acid (M9Cas) medium; both media contained glucose (0.2%, *w*/*v*). Briefly, the cultivation was performed in flasks (200 mL of culture in a 750 mL flask) on a shaker at 37 °C for 20 h. Purification of HBc-preS1 VLPs was performed with a combination of ion-exchange chromatography (IEX) and gel filtration (GF). A standard 2 g portion of frozen (−20 °C) cells was used for ultrasonic disintegration and obtained crude lysate was clarified at 10,000 rpm (13,000× *g*) for 30 min at +4 °C. The obtained suspension was first loaded on a Fractogel® EMD DEAE (M) (Merck, Burlington, Massachusetts, USA) column and fractions containing target protein were concentrated by tangential filtration using a 500 kD cartridge and obtained concentrate was further loaded on a Sepharose 4 Fast Flow (FF) (Cytiva Europe GmbH, Turku, Finland) column. SDS-polyacrylamide gel electrophoresis (SDS-PAGE, shortly PAGE) was used throughout the purification process to follow the presence of the target protein in fractions. The presence of VLPs in fractions was followed by native agarose gel electrophoresis (NAGE, see Section 2.5). The purity of protein samples was evaluated by PAGE.

### 2.5. Characterization of HBc-preS1 VLPs

For VLP detection, samples were subjected to (NAGE) using 1% UltraPure agarose (Thermo Fisher Scientific, Waltham, MA, USA) in TBE buffer. Ethidium bromide (EtBr, 5 µL of a 10 mg/mL stock in 100 mL of PBS) was used for staining NAGE gels and Coomassie Brilliant Blue R-250 (60 mg/L in 10% acetic acid) was used for the staining of PAGE and NAGE gels. All chemicals were from Sigma-Aldrich (St. Louis, MO, USA).

The purity of final HBc-preS1 preparations as VLPs was evaluated by PAGE gels (15%) stained with Coomassie Brilliant Blue R-250. Anti-HBc mAb 13C9 [65] and/or anti-preS1 mAb MA18/7 [9] at 1:1000 dilution were used for the Western blot of HBc-preS1 VLPs after PAGE. The morphology of VLP preparations was analyzed by transmission electron microscopy (EM) and the homogeneity of particles in VLP preparation by dynamic light scattering (DLS), as described earlier [54].

### 2.6. The Antigenicity of the HBc-preS1 VLPs

For the competitive ELISA, 96-well microplates were coated with 100 µL of preS1(20–47) peptide solution (10 µg/mL) in 50 mM sodium carbonate buffer, pH 9.6 per well and incubated overnight at 4 °C. After blocking with phosphate-buffered saline (PBS) containing 1% BSA for 1 h at RT, 50 µL aliquots of serial dilutions of test proteins and 50 µL of the anti-preS1 mAb MA18/7 (dilution 1:500) [9] were added to the wells simultaneously. Plates were incubated at 37 °C for 1 h, then washed four times with Tween-20 containing (0.05%) PBS. Thereafter, 100 μL of horseradish peroxidase-conjugated anti-mouse antibody (Sigma-Aldrich St. Louis, MO, USA) was added to wells at a 1:10,000 dilution and incubated at 37 °C for 1 h. After washing the plates four times as before, OPD substrate (Sigma–Aldrich St. Louis, MO, USA) was added to develop the color. The percent inhibition (I%) of antibody binding by the competing protein was calculated as follows: I% = [(OD492 test sample—OD492 of negative control)/(OD492 of positive control—OD492 negative control)] × 100. The molar amount of the protein necessary for 50% inhibition (I50) was calculated.

### 2.7. Immunogenicity of the HBc-preS1 VLPs

Immunization of BALB/c mice with HBc-preS1 VLPs was performed as described before in [52] with the permission of the Latvian Animal Protection Ethics Committee (Permission No. 61/12.05.2014). Five animals in each group were immunized subcutaneously with 25 µg of VLPs in PBS formulated with 250 µg of Alhydrogel in a total volume of 0.2 mL per mouse at days 0, 14, and 28. Sera injected with Alhydrogel only animals were used as negative controls. Anti-HBc and anti-preS1 titers in the sera were detected with direct ELISA. The recombinant full-length HBc protein (as VLPs) or preS1 peptide (20–47 aa), both at 10 μg/mL, was used for plate coating. The end-point titers were defined as the highest mAb dilution that resulted in an absorbance value three times greater than that of the negative control.

T-cell proliferation tests were performed as described in [52] in the lymphocytes of the mice immunized with the HBc-preS1 VLPs. Spleens were collected on day 42 post-immunization and splenocytes from the mice of each group were pooled. In vitro stimulation was performed using full-length HBc1-183 protein at 1.0 μg/mL and 10 μg/mL concentration. Concanavalin A (ConA) at 4 µg/mL was used as a positive control. Results of T-cell proliferation were presented as stimulation indexes (SI), which were calculated as a ratio of mean cpm obtained in the presence and absence of HBc.

### 2.8. CpG Oligodeoxynucleotide Packaging

Synthetic oligodeoxynucleotide (ODN) 1668 (5′-tccatgacgttcctgatgct-3′) is the B-class unmethylated CpG dinucleotide specific for mouse Toll-like receptor 9 (TLR9), strongly activates B cells but weakly IFN-α secretion. ODN 1668 was obtained from InvivoGen (Toulouse, France) and it was tested here with 183preS1(20–47) VLPs for the packaging. ODN packaging was performed according to the method described in [66] with the use of RNase and urea. For packaging the following mix was prepared: 50 µg/12,5 µL of VLPs in PBS + 100 µg/10 µL RNaseA (Thermo Fisher Scientific, cat. No R1253) + 50 µL 1 M urea in water + 7 µg/30 µL ODN 1668 in water. The packaging mix was incubated at room temperature overnight.

### 2.9. The 3D Modeling of HBc-preS1 VLPs

The VIPERdb v3.0 (http://viperdb.scripps.edu, accessed on 1 May 2022) [67] was used to create maps of HBc VLPs and 3DJIGSAW protein modeling program [68] was applied for the prediction of three-dimensional structures of HBc-preS1 VLPs. The UCSF Chimera Version 1.16 package from the Resource for Biocomputing, Visualization, and Informatics at the University of California, San Francisco [49] was utilized for the production of molecular graphics images.

## 3. Results

### 3.1. Modeling of HBc-preS1 Structures

The externally exposed region MIR within the c/e1 epitope located on the tip of the spikes of HBc (Figure 1) was chosen for the insertion of two preS1 fragments: one, short (aa 20–47) of the preS1, and a second long, preS1phil fragment, (aa 12–60 + 89–119) representing the preS1 sequence with the deleted hydrophobic region (aa 61–88) [60] (Figure 2A). Five HBc gene variants ending at aa positions 163, 167, 171, 178, and 183 of HBV were used for the preS1 insertions, generating in total ten HBc-preS1 chimeric proteins (Figure 2B).

**Figure 3:**
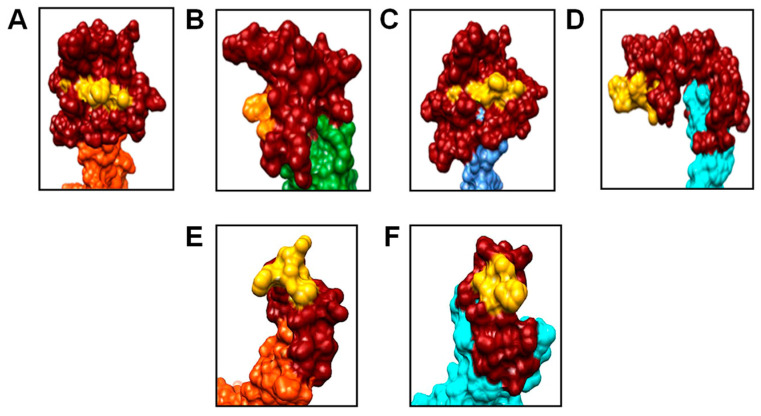
Structure modeling of 183preS1 VLPs. (**A**–**D**) preS1phil insertion in four possible HBc monomers A, B, C, and D, and (**E**,**F**) preS1(20–47) insertion in monomers A and D. HBc monomers are colored: A—red-orange, B—green, C—blue, and F—(cyan). PreS1 insertions are colored dark red with the DPAFR shown in yellow. Note: inserted preS1 fragments are enlarged and in orientation to highlight the position of the DPAFR within the insert. Data were downloaded from the VIPERdb v3.0 (http://viperdb.scripps.edu, accessed on 1 May 2022) and visualized using Chimera Version 1.16 software [48].

The possible three-dimensional organization of the preS1 fragments within chimeric 183HBc-preS1 VLPs as predicted by 3D-JIGSAW is shown in Figure 3. This program creates three-dimensional maps grounded on homologies of highly resolved structures [64]. Figure 3 depicts the modeling results of the modified MIR region for all four possible HBc monomers (conformers [42]) A–D exposing the long preS1phil fragment (Figure 3A–D) and for two monomers A and D exposing short preS1(20–47) (Figure 3E,F).

### 3.2. The Expression of HBc-preS1 Proteins

The expression level of the target protein in 10 individual transformed cell clones was compared before the selection of the best clone of transformed cells for further use. The combination of host strain (*E. coli* K802 or *E. coli* BL21) and cultivation medium (2xTYP or M9+Cas [54]) was experimentally found for each of ten HBc-preS1 proteins by the cultivation of transformed cells first in tubes (with 5 mL of culture in 15 mL tubes). The 5 mL culture served later as the seed culture for scale-up cultivation in flasks to obtain several grams of biomass (see Materials and Methods, Section 2.4). *E. coli* K802 was found optimal for HBc-preS1constructs based on the full-length HBc protein but *E. coli* BL21—for other constructs based on shortened HBc variants as a carrier protein. The lowest cell density was found for cultures expressing HBc-preS1 proteins based on 183 aa-long full-length HBc. Thus, the typical OD_540_ for cell cultures expressing 183preS1 proteins reached: approximately 6 OD units in 2TYP medium, and approximately 4 OD units in M9Cas medium, approximately 8 for cell cultures expressing 161-preS1, 163preS1, 171preS1, and 178preS1 proteins in 2xTYP medium, and approximately 6 in M9Cas medium. The expression level of different HBc-preS1 proteins varied within the range of 7–10% of total cellular protein with an overall higher level for fusion proteins based on shortened HBc and with shorter preS1(20–47) insert. It was found that in all investigated cases, HBc-preS1 proteins were able to form capsid-like structures (VLPs) within expressing cells.

### 3.3. Purification of HBc-preS1 Proteins as VLP

**Figure 4:**
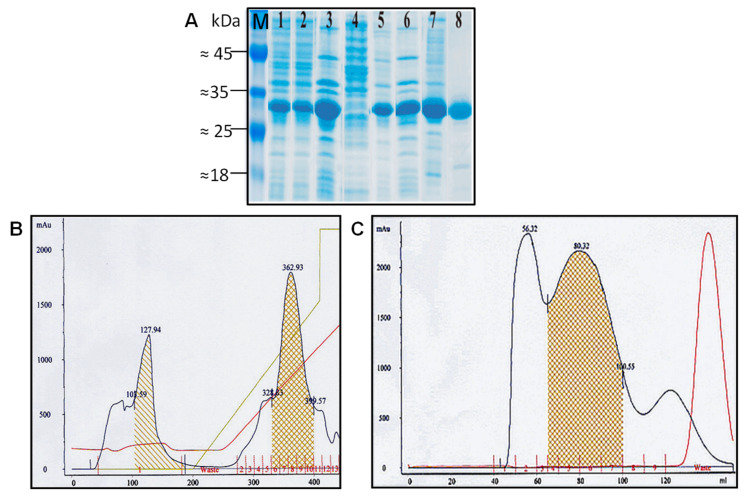
The purification of 183preS1phil VLPs. (**A**) SDS-PAGE of fractions from the entire purification process, staining with Coomassie Brilliant Blue G-250: M - Pierce™ Prestained Protein MW Marker (cat N 26612, Thermo Fisher Scientific Baltics UAB, Vilnius, Lithuania), lane 1—crude cell lysate (after French press), lanes 2, 3—extraction with 1M urea: lane 2—supernatant, lane 3—debris; lane 4—supernatant after precipitation with 35% ammonium sulfate (AS); lanes 5, 6—extraction with 1M urea of the precipitate after 35% AS: lane 5—soluble part, lane 6—insoluble part; lane 7—central peak fractions from Fractogel EMD DEAE (see second peak on (**B**)pooled fractions for GF are shown cross-striped and colored in brown); lane 8—central peak fractions from GF on Sepharose 4 FF (see (**C**), pooled fractions are shown crossed-striped and colored in brown. (**B**) Fractogel EMD DEAE chromatography; (**C**) GF on Sepharose 4 FF. In (**B**,**C**) X-axis show mL, Y-axis—optical density (OD, A_254_) in adsorption units (mAu); analyzed fractions are shown under the peaks and are red colored; blue line shows OD, red line shows conductivity and green line shows NaCl gradient at elution study. Left part in (**B**) shows that the capacity of the column was exceeded and part of target protein was not loaded (shown striped).

Target proteins were purified as VLPs from the soluble protein fraction of disintegrated cells after extraction with urea, followed by ammonium sulfate fractionation, and two-step chromatography, using IEX on Fracto DEAE as a first step followed by GF on 4 FF Sepharose. Purification of 183preS1phil as an example is shown in Figure 4.

### 3.4. Characterization of HBc-preS VLP

**Figure 5:**
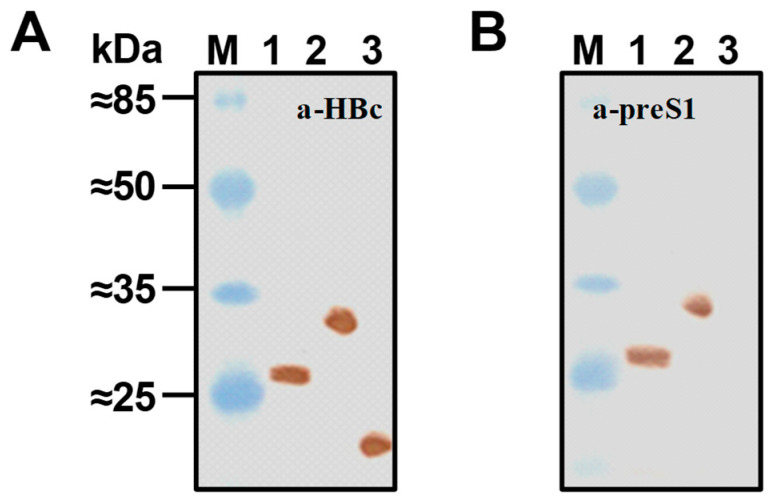
WB of 183preS1(20–47) and 183preS1phil proteins. (**A**) Blot with anti-HBc mAb13C9 [66]; (**B**) blot with anti-preS1 mAb MA18/7 [9]. Lane 1—183preS1(20–47), lane 2—183preS1phil, lane 3—HBc183. M—Prestained Protein Molecular Weight Marker (26612, Pierce™). Normalized amounts of VLPs (10 μg) were used for SDS-PAGE.

The identity of HBc-preS1 proteins after their purification as VLPs was verified by Western blot (WB) using anti-HBc mAb 13C9 [65] and anti-preS1 mAb MA18/7 [8]. Representative WB for 183preS1(20–47) and 183preS1phil proteins purified as VLPs is shown in Figure 5.

**Figure 6:**
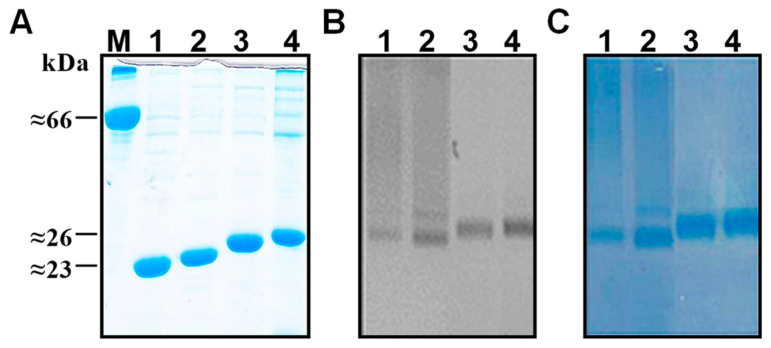
SDS-PAGE and NAGE of HBc-preS1(20–47) VLPs preparations. Loaded proteins: M—BSA, lane 1—163preS1(20–47), lane 2—167preS1(20–47), lane 3—178preS1(20–47), and lane 4—183preS1(20–47). (**A**) 15% SDS-PAGE, (**B**,**C**) 1% NAGE in TBE buffer of the same VLP samples: (**B**) EtBr staining, (**A**,**C**) Coomassie Brilliant Blue R-250 staining. Note: 171preS1(20–47) not shown.

NAGE gels revealed the presence of nucleic acids in VLPs of all kinds of HBc-preS1 VLPs. Figure 6 shows representative PAGE and NAGE for the part of HBc-preS1 proteins, with the short preS1(20–47) as an insert.

**Figure 7:**
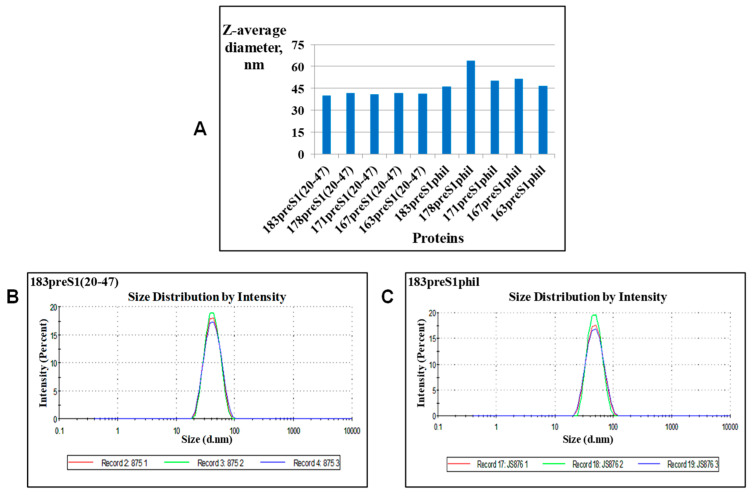
DLS analysis of the HBc-preS1 VLPs preparations. (**A**) Z-average of VLPs for all investigated VLP preparations. (**B**,**C**) Size distribution for VLPs of 183preS1(20–47) and 183preS1phil proteins, respectively.

DLS was used as a method to characterize the medium size of VLPs in the preparations (Z-average) along with homogeneity of particles (presence of aggregates), and polydispersity, expressed as polydispersion index (Pdi). The Z-average in pooled central GF peak fraction from GF as the final step in VLP purification (see Figure 4C) among all 10 different HBc-preS1 VLP preparations was, with the exception of 178preS1phil variant, in the range of 40–45 nm, (Figure 7A). The presence of aggregates was not revealed in any of the ten preparations of investigated VLP variants (see Figure 7B,C for two representative constructs—183preS1(20–47) and 183preS1phil). Pdi for all investigated VLP preparations was below 0.2 (0.08–0.168). Pooled central GF peak fractions contained at least 0.5 mg/mL of protein in all cases, with the content of target protein over 90% as estimated by SDS-PAGE. The yield of VLPs for all investigated HBc-preS1 protein variants was in the range of 20–30 mg/g of fresh biomass.

**Figure 8:**
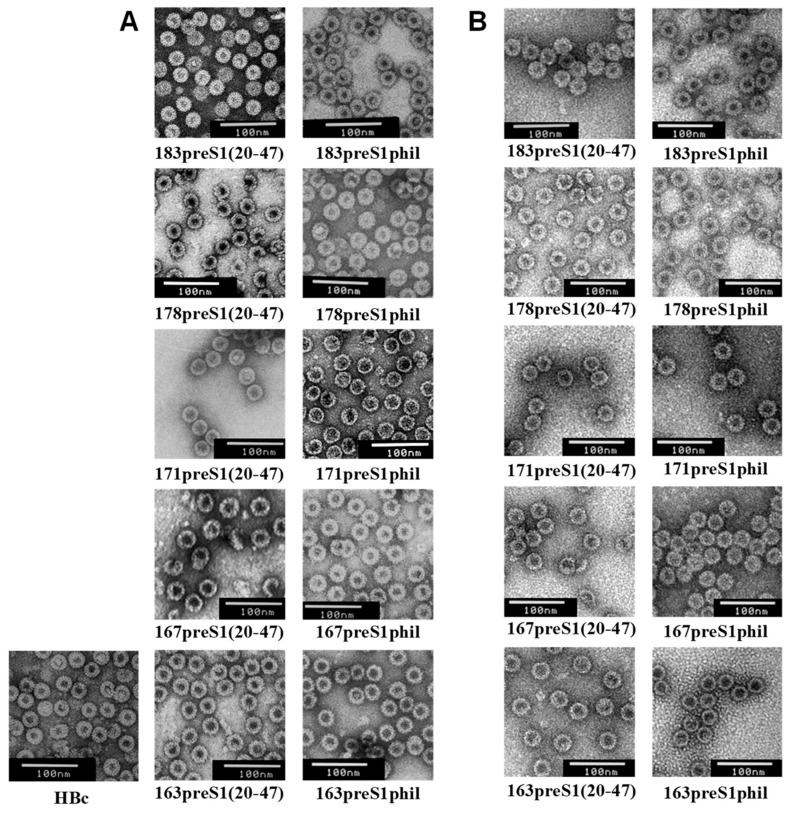
EM of HBc-preS1 VLP preparations. (**A**) Original preparations (years 2016—2018); (**B**) the same samples in the year 2021. Scale bar, 100 nm.

VLP quality evaluation was performed by visualizing the particles by transmission EM using a negative staining protocol. VLPs formed by preS1-HBc proteins with a short preS1(20–47) insert were of the size similar to the VLPs of non-chimeric HBc and slightly bigger with a longer preS1phil insert used for the construction of HBc-preS1 proteins (Figure 8). We did not observe the instability of VLPs after storage in 50% glycerol at −18 °C for several years (Figure 8B).

### 3.5. Accessibility of the preS1 Epitope in the HBc-preS1 VLPs to the mAb MA18/7

**Figure 9:**
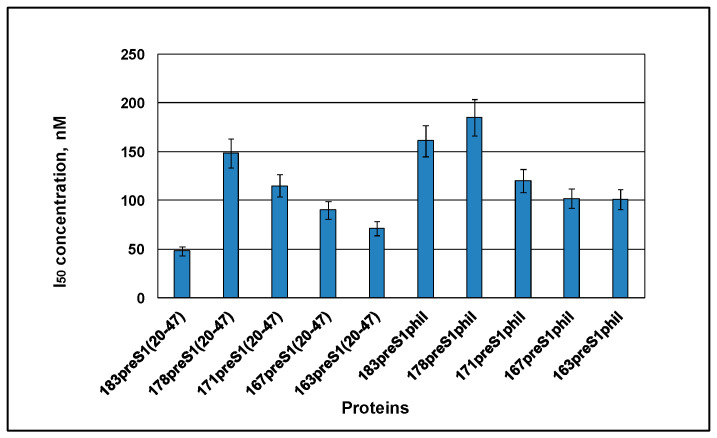
The preS1 antigenicity of the HBc-preS1 VLPs. The VLP concentration necessary and sufficient to inhibit 50% of the binding of mAb MA18/7 [9] to the 20–47 peptide on the support during the competitive ELISA is shown. Tests were done in triplicate.

Accessibility of the inserted preS1 epitope to a specific antibody was characterized by competitive ELISA using mAb MA18/7 [9]. The 50% inhibitory concentration (Figure 9) was in the range of 48–185 nM for different HBc-preS1 constructs. The competition lag of preS1phil-containing constructs compared to constructs with preS1(20–47) is possibly due to the remarkably longer inserted preS1 sequence (80 aa).

### 3.6. Immunogenicity of HBc-preS1 VLPs

**Figure 10:**
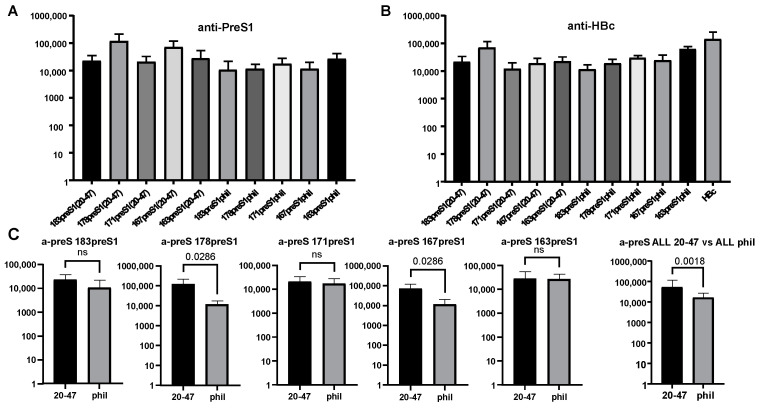
Immunogenicity of HBc-preS1 proteins. BALB/c mice were immunized with VLPs of HBc-preS1 proteins shown under bars in (**A**,**B**). (**A**) Anti-preS1 response and (**B**) anti-HBc response. (**C**) Statistical analysis of antibody response (Mann–Whitney U test). The type of HBc vector (with 183, 178, 171, 167, 163 aa of HBc) used in fusion constructs is shown on the top of the bars and preS1 fragment (20–47 or preS1phil) used in fusion proteins is shown under the bars. The last two bars show the comparison of all five constructs with preS1(20–47) insertion to all five constructs with preS1phil insertion. p-value is indicated on the top of the bars; ns = not significant.

Anti-HBc response in the control group immunized with unmodified HBc VLPs (without any insertion) reached the titer of anti-HBc to the level 1:164,025. However, the anti-HBc titers for HBc constructs with MIR insertions were substantially decreased, with some exception for construct 178preS1(20–47) (Figure 10B). As for anti-preS1(20–47) response, there was a tendency of higher response for constructs with short preS1(20–47) insertions rather than with preS1phil insertions; however, the differences were not always significant (Figure 10C).

**Figure 11:**
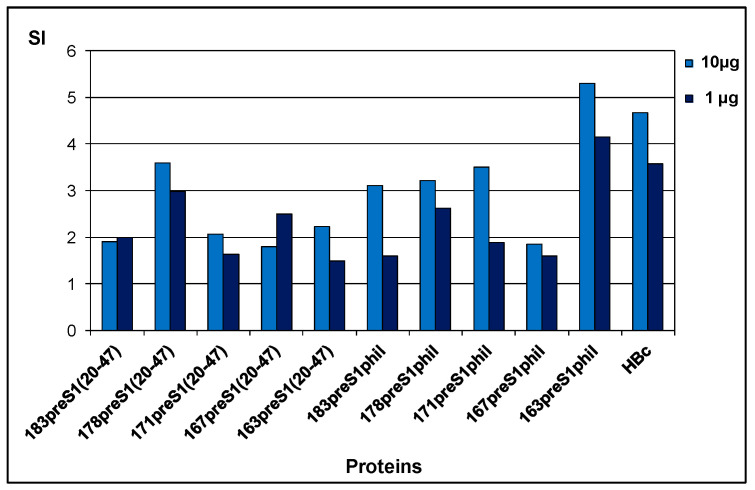
T-cell proliferation in splenocytes of BALB/c mice immunized with HBc-preS1 VLPs. Stimulation indexes (SI) were calculated after stimulation with HBc183 (1 µg and 10 µg) on day 42 after the first immunization. Types of VLPs used for immunization are shown under the bars.

T cells were stimulated in vitro using two doses of full-length 183HBc (1 µg and 10 µg), and appropriate SI were determined. SI values of 2.0 and above were considered positive. Clear proliferation was detected for all immunization cases (Figure 11). For the constructs with a preS1(20–47) insert, the best proliferation effect was found in the group of animals immunized with the VLPs formed by a 178 aa-long HBc vector (178preS1(20–47)), and for constructs with preS1phil insert—in the group of animals immunized with a 163 aa-long HBc vector (163preS1phil).

### 3.7. Packaging of Oligonucleotide ODN 1668 in Chimeric HBc-preS1 VLPs

**Figure 12:**
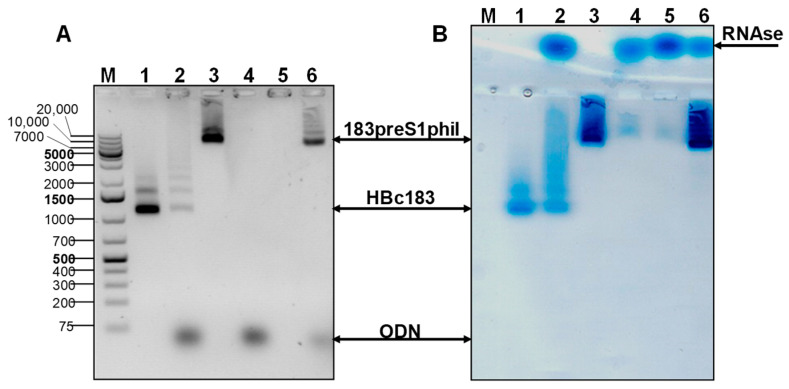
Packaging of ODN 1668 in the VLPs of 183pS1phil protein in the conditions of treatment with RNase and urea. (**A**) EtBr stained NAGE, of HBc and 183preS1phil VLPs before and after treatment: M—marker (GeneRuller 1 kb DNA Ladder (SM1331, Thermo Fisher Scientific™, Waltham, MA, USA), 1—full-length HBc VLPs (HBc183) without treatment, 2—HBc183 treated with RNaseA and added ODN 1668, 3—183preS1phil VLPs without treatment, 4—183preS1phil VLPs treated with RNaseA and added ODN 1668, 5—183preS1phil VLPs treated with RNaseA and urea, without ODN 1668, and 6—183preS1phil VLPs treated with RNaseA and urea with added ODN 1668. (**B**) The same NAGE gel, stained with Coomassie Brilliant Blue G250. Note: lanes 4 and 5 show the absence of protein which is explained by the precipitation of empty VLPs after treatment with RNase; the supernatants of centrifuged (10 min at 13,000× *g*) samples of treated VLPs were loaded on a gel.

The ability of chimeric HBc-preS1 VLPs to incorporate immunostimulating CpG sequences was tested with the use of synthetic ODN 1668 and VLPs formed by 183preS1phil protein. To eliminate from VLPs incorporated RNA of host origin, treatment of VLPs with RNaseA was performed in the presence of urea and ODN 1668 (Materials and Methods, Section 2.7). As shown in Figure 12, the packaging was successful (Figure 12A, line 6). It was observed that although RNA can be eliminated with RNase treatment alone, effective packaging is ensured only when also in the presence of urea (compare lanes 4 and 6 in Figure 11A); however, without the urea, some packaging was observed in VLPs of unmodified HBc (Figure 12, lane 2). Treated VLPs (with RNaseA and with RNaseA + urea) lose their ability to move in gel and treated VLP material stays on the start position (see some staining signal at the start of lanes 2, 4, and 5 in Figure 12). RNaseA in NAGE moves in the opposite direction as seen in Figure 12B.

## 4. Discussion

Currently available HBV vaccines are for prophylactic use, being ineffective for the treatment of chronic HBV carriers. These vaccines are based on the pure S protein of the HB antigen or are the combination of the S with the M and/or L forms of HBs. Although these vaccines have demonstrated their effectiveness in the vaccination of newborns, they are less effective in certain groups of people such as aged and obese people or are ineffective in chronic HBV carriers.

This work aimed to generate the recombinant VLPs presenting in two HBV antigens—preS1 for B-cell immunogenicity and HBc for T-cell immunogenicity: preS1 contains the virus-neutralizing epitope and HBc is the source of HBV-specific CTL epitopes. Thus, the rationale of our study lies within the findings that the combination of preS1 sequences and HBc in the particular HBV vaccine candidate not only extends its protective effect but also, due to the presence of HBc, compensates for the lack of T-cell immunogenicity.

The exposure of the preS1 epitopes on the HBc VLPs has a long history and was generally performed by our group [9,69,70,71,72], Ken Murray’s group [73,74,75], David Milich and Florian Schödel’s group [76,77,78], and Xinchun Chen’s group [79]. Later, Matti Sällberg’s team added additional reasons for the expected efficiency of the HBc-preS vaccines [80]. In this resumptive study, we summarized our research on the construction of HBc-preS1 VLPs.

Here, two fragments of the preS1 region of the hepatitis B virus, genotype D1, subtype ayw2 [47] have been used for insertion in HBc protein: (i) the “pure” preS1 epitope corresponding to aa 20–47 of the preS1, and (ii) a preS1phil fragment, aa 12–60 + 89–119, representing the preS1 sequence with a deleted hydrophobic (aa 61–88) region [60]. Both preS1 fragments contain a linear preS1 epitope 31-DPAFR-35 [9,10] which is recognized by highly specific mAb MA18/7 [8]. Selected preS1 sequences were inserted in the MIR of HBc protein using a set of full-length and C-terminally truncated variants of HBc, and the immunogenicity of ten different HBc-preS1 VLP constructs was compared in mice. The production level of the different HBc-preS1 fusion proteins in *E. coli* cells was remarkable (reaching 7–10% of total cell protein in crude cell lysate), allowing us the development of non-sophistical purification protocol and to obtain high-quality VLPs suitable for further immunological evaluation.

As for B-cell immunogenicity, both preS1(20–47) and preS1phil containing VLPs induced a significant anti-preS1 response (Figure 10A) along with the decreased response to the carrier HBc (Figure 10B), caused by damaged MIR within HBc formed VLPs. All variants of the HBc-preS1 VLPs competed well in ELISA with preS1 peptide coated on the plate for the MA18/7 antibodies (Figure 9). These data confirm that the major preS1 epitope DPAFR is exposed and localized correctly on the surface of the chimeric particles, and that conformation of preS1 in our constructs is native like. Additionally, it can be concluded that the N- and C-terminals surrounding of the major immunodominant DPAFR epitope are not important for the correct exposure of the preS1 epitope for induction of the preS1-specific humoral response.

As for T-cell immunogenicity, the T-cell proliferation index was found to be high for all investigated constructs; however, the SI varied significantly among the different constructs with the highest SI for 163preS1phil as a representative of the constructs with long preS1phil insert, and 178preS1(20–47) as a representative of constructs with a short preS1(20–47) insert (Figure 11).

As the antibodies elicited against the preS1 epitope should be strongly virus neutralizing [7], we suggest that recombinant HBc VLPs bearing preS1 sequences may serve as real prototypes for the creation of a combined therapeutic/prophylactic HBV vaccine according to the criteria formulated by Gerlich [81]. The sound biotechnological background of the production and purification of HBc-preS1 VLPs allowed us to obtain tens of mg of highly purified chimeric VLPs from the amount of fresh biomass by standardized protocol and this factor underlines the possible practical application of the elaborated HBc-preS1 VLPs as the candidates for further immunological investigations aimed to the development of the universal prophylactic/therapeutic vaccine.

Packaging of a selected oligonucleotide inside the VLPs has also been demonstrated in the case of full-length HBc bearing a long preS1phil insert after removal of intact RNA (Figure 12).

## 5. Patents

A. Dišlers, I. Petrovskis, I. Liekniņa, I. Berza, J. Bogans, I. Akopjana, I. Sominska, P. Pumpens Latvian Patent C12N15/71 21.06.2013. Expression system for obtaining of HBc-pres1 virus-like particles.

I. Liekniņa, I. Petrovskis, I. Sominska, J. Bogans, I. Akopjana, P. Pumpens, A. Dišlers Latvian Patent C12N15/70 20.12.2017. Method for obtaining empty and packed with nucleic acids capsids of virus-like particles of HBc-protein.

## Data Availability

Not applicable.

## References

[1] Chen H.L., Lin L.H., Hu F.C., Lee J.T., Lin W.T., Yang Y.J., Huang F.C., Wu S.F., Chen S.C., Wen W.H. (2012). Effects of maternal screening and universal immunization to prevent mother-to-infant transmission of HBV. Gastroenterology.

[2] Shouval D., Roggendorf H., Roggendorf M. (2015). Enhanced immune response to hepatitis B vaccination through immunization with a Pre-S1/Pre-S2/S vaccine. Med. Microbiol. Immunol..

[3] Suk-Fong Lok A. (2018). Hepatitis B Treatment: What We Know Now and What Remains to Be Researched. Hepatol. Commun..

[4] Backes S., Jäger C., Dembek C.J., Kosinska A.D., Bauer T., Stephan A.S., Dišlers A., Mutwiri G., Busch D.H., Babiuk L.A. (2016). Protein-prime/modified vaccinia virus Ankara vector-boost vaccination overcomes tolerance in high-antigenemic HBV-transgenic mice. Vaccine.

[5] Whitacre D.C., Peters C.J., Sureau C., Nio K., Li F., Su L., Jones J.E., Isogawa M., Sallberg M., Frelin L. (2020). Designing a therapeutic hepatitis B vaccine to circumvent immune tolerance. Hum. Vaccines Immunother..

[6] Maeng C.Y., Ryu C.J., Gripon P., Guguen-Guillouzo C., Hong H.J. (2000). Fine mapping of virus-neutralizing epitopes on hepatitis B virus PreS1. Virology.

[7] Bremer C.M., Sominskaya I., Skrastina D., Pumpens P., El Wahed A.A., Beutling U., Frank R., Fritz H.J., Hunsmann G., Gerlich W.H. (2011). N-terminal myristoylation-dependent masking of neutralizing epitopes in the preS1 attachment site of hepatitis B virus. J. Hepatol..

[8] Heermann K.H., Goldmann U., Schwartz W., Seyffarth T., Baumgarten H., Gerlich W.H. (1984). Large surface proteins of hepatitis B virus containing the pre-S sequence. J. Virol..

[9] Sominskaya I., Pushko P., Dreilina D., Kozlovskaya T., Pumpen P. (1992). Determination of the minimal length of preS1 epitope recognized by a monoclonal antibody which inhibits attachment of hepatitis B virus to hepatocytes. Med. Microbiol. Immunol..

[10] Germaschewski V., Murray K. (1995). Screening a monoclonal antibody with a fusion-phage display library shows a discontinuity in a linear epitope within PreS1 of hepatitis B virus. J. Med. Virol..

[11] Glebe D., Aliakbari M., Krass P., Knoop E.V., Valerius K.P., Gerlich W.H. (2003). Pre-s1 antigen-dependent infection of Tupaia hepatocyte cultures with human hepatitis B virus. J. Virol..

[12] Brian P. Dunleavy Public Health Watch: New HBV Vaccine Shows Promise in Phase 3 Trial, 25 May 2021. https://www.contagionlive.com/view/public-health-watch-new-hbv-vaccine-shows-promise-in-phase-3-trial.

[13] Hellström U.B., Madalinski K., Sylvan S.P. (2009). PreS1 epitope recognition in newborns after vaccination with the third-generation Sci-B-Vac vaccine and their relation to the antibody response to hepatitis B surface antigen. Virol. J..

[14] Krawczyk A., Ludwig C., Jochum C., Fiedler M., Heinemann F.M., Shouval D., Roggendorf M., Roggendorf H., Lindemann M. (2014). Induction of a robust T- and B-cell immune response in non- and low-responders to conventional vaccination against hepatitis B by using a third generation PreS/S vaccine. Vaccine.

[15] Murray K., Bruce S.A., Hinnen A., Wingfield P., van Erd P.M., de Reus A., Schellekens H. (1984). Hepatitis B virus antigens made in microbial cells immunise against viral infection. EMBO J..

[16] Iwarson S., Tabor E., Thomas H.C., Goodall A., Waters J., Snoy P., Shih J.W., Gerety R.J. (1985). Neutralization of hepatitis B virus infectivity by a murine monoclonal antibody: An experimental study in the chimpanzee. J. Med. Virol..

[17] Murray K., Bruce S.A., Wingfield P., van Eerd P., de Reus A., Schellekens H. (1987). Protective immunisation against hepatitis B with an internal antigen of the virus. J. Med. Virol..

[18] Roos S., Fuchs K., Roggendorf M. (1989). Protection of woodchucks from infection with woodchuck hepatitis virus by immunization with recombinant core protein. J. Gen. Virol..

[19] Schödel F., Neckermann G., Peterson D., Fuchs K., Fuller S., Will H., Roggendorf M. (1993). Immunization with recombinant woodchuck hepatitis virus nucleocapsid antigen or hepatitis B virus nucleocapsid antigen protects woodchucks from woodchuck hepatitis virus infection. Vaccine.

[20] Menne S., Maschke J., Tolle T.K., Lu M., Roggendorf M. (1997). Characterization of T-cell response to woodchuck hepatitis virus core protein and protection of woodchucks from infection by immunization with peptides containing a T-cell epitope. J. Virol..

[21] Lu M., Hilken G., Kruppenbacher J., Kemper T., Schirmbeck R., Reimann J., Roggendorf M. (1999). Immunization of woodchucks with plasmids expressing woodchuck hepatitis virus (WHV) core antigen and surface antigen suppresses WHV infection. J. Virol..

[22] Lu M., Roggendorf M. (2001). Evaluation of new approaches to prophylactic and therapeutic vaccinations against hepatitis B viruses in the woodchuck model. Intervirology.

[23] Zhang E., Kosinska A.D., Ma Z., Dietze K.K., Xu Y., Meng Z., Zhang X., Wang J., Wang B., Dittmer U. (2015). Woodchuck hepatitis virus core antigen-based DNA and protein vaccines induce qualitatively different immune responses that affect T cell recall responses and antiviral effects. Virology.

[24] Miller D.S., Halpern M., Kotlarski I., Jilbert A.R. (2006). Vaccination of ducks with a whole-cell vaccine expressing duck hepatitis B virus core antigen elicits antiviral immune responses that enable rapid resolution of de novo infection. Virology.

[25] Boudewijns R., Ma J., Neyts J., Dallmeier K. (2021). A novel therapeutic HBV vaccine candidate induces strong polyfunctional cytotoxic T cell responses in mice. JHEP Rep..

[26] Pyrski M., Rugowska A., Wierzbiński K.R., Kasprzyk A., Bogusiewicz M., Bociąg P., Samardakiewicz S., Czyż M., Kurpisz M., Pniewski T. (2017). HBcAg produced in transgenic tobacco triggers Th1 and Th2 response when intramuscularly delivered. Vaccine.

[27] Pyrski M., Mieloch A.A., Plewiński A., Basińska-Barczak A., Gryciuk A., Bociąg P., Murias M., Rybka J.D., Pniewski T. (2019). Immunization with Plant-Derived HBcAg as a Potential Therapeutic Vaccine against Chronic Hepatitis B. Vaccines.

[28] Sanada T., Yamamoto N., Kayesh M.E.H., Tsukiyama-Kohara K., Hasegawa H., Miyazaki T., Takano J.I., Shiogama Y., Yasutomi Y., Goh Y. (2019). Intranasal vaccination with HBs and HBc protein combined with carboxyl vinyl polymer induces strong neutralizing antibody, anti-HBs IgA, and IFNG response. Biochem. Biophys. Res. Commun..

[29] Spellman M., Martin J.T. (2011). Treatment of chronic hepatitis B infection with DV-601, a therapeutic vaccine. J. Hepatol..

[30] Li J., Ge J., Ren S., Zhou T., Sun Y., Sun H., Gu Y., Huang H., Xu Z., Chen X. (2015). Hepatitis B surface antigen (HBsAg) and core antigen (HBcAg) combine CpG oligodeoxynucletides as a novel therapeutic vaccine for chronic hepatitis B infection. Vaccine.

[31] Betancourt A.A., Delgado C.A., Estévez Z.C., Martínez J.C., Ríos G.V., Aureoles-Roselló S.R., Zaldívar R.A., Guzmán M.A., Baile N.F., Reyes P.A. (2007). Phase I clinical trial in healthy adults of a nasal vaccine candidate containing recombinant hepatitis B surface and core antigens. Int. J. Infect. Dis..

[32] Lobaina Y., Michel M.L. (2017). Chronic hepatitis B: Immunological profile and current therapeutic vaccines in clinical trials. Vaccine.

[33] Lopez M., Rodriguez E.N., Lobaina Y., Musacchio A., Falcon V., Guillen G., Aguilar J.C. (2017). Characterization of the size distribution and aggregation of virus-like nanoparticles used as active ingredients of the HeberNasvac therapeutic vaccine against chronic hepatitis B. Adv. Nat. Sci. Nanosci. Nanotechnol..

[34] Al Mahtab M., Akbar S.M.F., Aguilar J.C., Guillen G., Penton E., Tuero A., Yoshida O., Hiasa Y., Onji M. (2018). Treatment of chronic hepatitis B naïve patients with a therapeutic vaccine containing HBs and HBc antigens (a randomized, open and treatment controlled phase III clinical trial). PLoS ONE.

[35] Chain B.M., Myers R. (2005). Variability and conservation in hepatitis B virus core protein. BMC Microbiol..

[36] Birnbaum F., Nassal M. (1990). Hepatitis B virus nucleocapsid assembly: Primary structure requirements in the core protein. J. Virol..

[37] Seifer M., Standring D.N. (1994). A protease-sensitive hinge linking the two domains of the hepatitis B virus core protein is exposed on the viral capsid surface. J. Virol..

[38] Pumpens P., Grens E. (2016). The true story and advantages of the famous Hepatitis B virus core particles: Outlook 2016. Mol. Biol..

[39] Pumpens P., Pushko P. (2022). Order Blubervirales. Virus-like Particles: A Comprehensive Guide.

[40] Hatton T., Zhou S., Standring D.N. (1992). RNA- and DNA-binding activities in hepatitis B virus capsid protein: A model for their roles in viral replication. J Virol..

[41] Gallina A., Bonelli F., Zentilin L., Rindi G., Muttini M., Milanesi G. (1989). A recombinant hepatitis B core antigen polypeptide with the protamine-like domain deleted self-assembles into capsid particles but fails to bind nucleic acids. J. Virol..

[42] Wynne S.A., Crowther R.A., Leslie A.G. (1999). The crystal structure of the human hepatitis B virus capsid. Mol. Cell.

[43] Salfeld J., Pfaff E., Noah M., Schaller H. (1989). Antigenic determinants and functional domains in core antigen and e antigen from hepatitis B virus. J. Virol..

[44] Sällberg M., Rudén U., Wahren B., Noah M., Magnius L.O. (1991). Human and murine B-cells recognize the HBeAg/beta (or HBe2) epitope as a linear determinant. Mol. Immunol..

[45] Pumpens P., Grens E. (2001). HBV core particles as a carrier for B cell/T cell epitopes. Intervirology.

[46] Pumpens P., Ulrich R., Sasnauskas K., Kazaks A., Ose V., Grens E., Khudyakov Y. (2008). Construction of novel vaccines on the basis of the virus-like particles: Hepatitis B virus proteins as vaccine carriers. Medicinal Protein Engineering.

[47] Bichko V., Pushko P., Dreilina D., Pumpen P., Gren E. (1985). Subtype ayw variant of hepatitis B virus. DNA primary structure analysis. FEBS Lett..

[48] Pettersen E.F., Goddard T.D., Huang C.C., Couch G.S., Greenblatt D.M., Meng E.C., Ferrin T.E. (2004). UCSF Chimera—A visualization system for exploratory research and analysis. J. Comput. Chem..

[49] Pushko P., Pumpens P., Grens E. (2013). Development of Virus-Like Particle Technology from Small Highly Symmetric to Large Complex Virus-Like Particle Structures. Intervirology.

[50] Zeltins A. (2013). Construction and characterization of virus-like particles: A review. Mol. Biotechnol..

[51] Borisova G.P., Kalis J.V., Dishler A.V., Pumpen P.P., Gren E.J., Tsibinogin V.V., Kukaine R.A. (1985). Expression of Human Hepatitis B-virus Core Antigen Gene Variants in *Escherichia coli*. Biopolym. Cell.

[52] Sominskaya I., Skrastina D., Petrovskis I., Dishlers A., Berza I., Mihailova M., Jansons J., Akopjana I., Stahovska I., Dreilina D. (2013). A VLP library of C-terminally truncated Hepatitis B core proteins: Correlation of RNA encapsidation with a Th1/Th2 switch in the immune responses of mice. PLoS ONE.

[53] Strods A., Ose V., Bogans J., Cielens I., Kalnins G., Radovica I., Kazaks A., Pumpens P., Renhofa R. (2015). Preparation by alkaline treatment and detailed characterisation of empty hepatitis B virus core particles for vaccine and gene therapy applications. Sci. Rep..

[54] Petrovskis I., Lieknina I., Dislers A., Jansons J., Bogans J., Akopjana I., Zakova J., Sominskaya I. (2021). Production of the HBc Protein from Different HBV Genotypes in *E. coli*. Use of Reassociated HBc VLPs for Packaging of ss- and dsRNA. Microorganisms.

[55] Freivalds J., Dislers A., Ose V., Pumpens P., Tars K., Kazaks A. (2011). Highly efficient production of phosphorylated hepatitis B core particles in yeast Pichia pastoris. Protein Expr. Purif..

[56] Krieger J., Stifter K., Riedl P., Schirmbeck R. (2018). Cationic domains in particle-forming and assembly-deficient HBV core antigens capture mammalian RNA that stimulates Th1-biased antibody responses by DNA vaccination. Sci. Rep..

[57] Krieger J., Riedl P., Stifter K., Roman-Sosa G., Seufferlein T., Wagner M., Schirmbeck R. (2019). Endogenously Expressed Antigens Bind Mammalian RNA via Cationic Domains that Enhance Priming of Effector CD8 T Cells by DNA Vaccination. Mol. Ther..

[58] Riedl P., Buschle M., Reimann J., Schirmbeck R. (2002). Binding immune-stimulating oligonucleotides to cationic peptides from viral core antigen enhances their potency as adjuvants. Eur. J. Immunol..

[59] Riedl P., Stober D., Oehninger C., Melber K., Reimann J., Schirmbeck R. (2002). Priming Th1 immunity to viral core particles is facilitated by trace amounts of RNA bound to its arginine-rich domain. J. Immunol..

[60] Skrastina D., Bulavaite A., Sominskaya I., Kovalevska L., Ose V., Priede D., Pumpens P., Sasnauskas K. (2008). High immunogenicity of a hydrophilic component of the hepatitis B virus preS1 sequence exposed on the surface of three virus-like particle carriers. Vaccine.

[61] Sominskaya I., Skrastina D., Dislers A., Vasiljev D., Mihailova M., Ose V., Dreilina D., Pumpens P. (2010). Construction and immunological evaluation of multivalent hepatitis B virus (HBV) core virus-like particles carrying HBV and HCV epitopes. Clin. Vaccine Immunol..

[62] Dishlers A., Skrastina D., Renhofa R., Petrovskis I., Ose V., Lieknina I., Jansons J., Pumpens P., Sominskaya I. (2015). The hepatitis B virus core variants that expose foreign C-terminal insertions on the outer surface of virus-like particles. Mol. Biotechnol..

[63] Soberόn X., Covarrubias L., Bolivar F. (1980). Construction and characterization of new cloning vehicles, IV. Deletion derivatives of pBR322 and pBR325. Gene.

[64] Ovchinnikov I.A., Sverdlov E.D., Tsarev S.A., Khodkova E.M., Monastyrskaia G.S. (1982). Direct expression of the gene of human leukocyte interferon F in *Escherichia coli* cells. Dokl. Akad. Nauk SSSR.

[65] Bichko V., Schodel F., Nassal M., Gren E., Berzinsh I., Borisova G., Miska S., Peterson D.L., Gren E., Pushko P. (1993). Epitopes recognized by antibodies to denatured core protein of hepatitis B virus. Mol. Immunol..

[66] Kazaks A., Balmaks R., Voronkova T., Ose V., Pumpens P. (2008). Melanoma vaccine candidates from chimeric hepatitis B core virus-like particles carrying a tumor-associated MAGE-3 epitope. J. Biotechnol..

[67] Reddy V.S., Natarajan P., Okerberg B., Li K., Damodaran K.V., Morton R.T., Brooks C.L., Johnson J.E. (2001). Virus particle explorer (VIPER), a website for virus capsid structures and their computational analyses. J. Virol..

[68] Bates P.A., Kelley L.A., MacCallum R.M., Sternberg M.J. (2001). Enhancement of protein modeling by human intervention in applying the automatic programs 3D-JIGSAW and 3D-PSSM. Proteins.

[69] Borisova G., Arya B., Dislers A., Borschukova O., Tsibinogin V., Skrastina D., Eldarov M.A., Pumpens P., Skryabin K.G., Grens E. (1993). Hybrid hepatitis B virus nucleocapsid bearing an immunodominant region from hepatitis B virus surface antigen. J. Virol..

[70] Borisova G., Borschukova O., Skrastina D., Dislers A., Ose V., Pumpens P., Grens E. (1999). Behavior of a short preS1 epitope on the surface of hepatitis B core particles. Biol. Chem..

[71] Fehr T., Skrastina D., Pumpens P., Zinkernagel R.M. (1998). T cell-independent type I antibody response against B cell epitopes expressed repetitively on recombinant virus particles. Proc. Natl. Acad. Sci. USA.

[72] Kazaks A., Borisova G., Cvetkova S., Kovalevska L., Ose V., Sominskaya I., Pumpens P., Skrastina D., Dislers A. (2004). Mosaic hepatitis B virus core particles presenting the complete preS sequence of the viral envelope on their surface. J. Gen. Virol..

[73] Stahl S.J., Murray K. (1989). Immunogenicity of peptide fusions to hepatitis B virus core antigen. Proc. Natl. Acad. Sci. USA.

[74] Shiau A.L., Murray K. (1997). Mutated epitopes of hepatitis B surface antigen fused to the core antigen of the virus induce antibodies that react with the native surface antigen. J. Med. Virol..

[75] Murray K., Shiau A.L. (1999). The core antigen of hepatitis B virus as a carrier for immunogenic peptides. Biol. Chem..

[76] Schödel F., Will H., Milich D.R., Brown F., Chanock R.M., Ginsberg H.S., Lerner R.A. (1991). Hybrid hepatitis-B virus core/pre-S particles expressed in live attenuated *Salmonellae* for oral immunization. Vaccines 91.

[77] Schödel F., Peterson D., Hughes J., Milich D.R. (1993). A virulent Salmonella expressing hybrid hepatitis B virus core/pre-S genes for oral vaccination. Vaccine.

[78] Schödel F., Kelly S.M., Peterson D.L., Milich D.R., Curtiss R. (1994). Hybrid hepatitis B virus core-pre-S proteins synthesized in avirulent Salmonella typhimurium and Salmonella typhi for oral vaccination. Infect. Immun..

[79] Chen X., Li M., Le X., Ma W., Zhou B. (2004). Recombinant hepatitis B core antigen carrying preS1 epitopes induce immune response against chronic HBV infection. Vaccine.

[80] Malik I.R., Chen A., Brass A., Ahlén G., Rahman M., Sällberg M., Qureshi J.A., Frelin L. (2012). A bi-functional hepatitis B virus core antigen (HBcAg) chimera activates HBcAg-specific T cells and preS1-specific antibodies. Scand. J. Infect. Dis..

[81] Gerlich W.H. (2017). Do we need better hepatitis B vaccines?. Indian J. Med. Res..

